# General Characterization of Properties of Ordered and Disordered Proteins by Wide-Line ^1^H NMR

**DOI:** 10.1021/acsomega.4c00517

**Published:** 2024-05-22

**Authors:** Mónika Bokor, Ágnes Tantos

**Affiliations:** † Institute for Solid State Physics and Optics, HUN-REN Wigner Research Centre for Physics, 1121 Budapest, Hungary; ‡ HUN-REN Research Centre for Natural Sciences, Institute of Enzymology, 1117 Budapest, Hungary

## Abstract

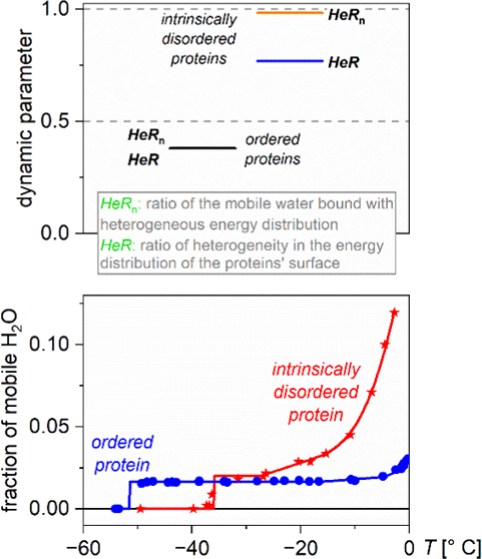

Wide-line ^1^H NMR is an efficient spectroscopic method to determine the disorder tendency of a protein. It directly measures the properties of the hydration shell of proteins, delivering exact and measurable values of their disorder/order content. A comparison is performed between several globular and disordered proteins. The common properties of the subzero mobile hydration water of these two groups were investigated. The amount of the mobile hydration water and the shape of the melting diagram at subzero temperatures together provide a possibility to distinguish globular proteins from disordered proteins. The shape of the melting diagram also gives information about the presence of secondary structural elements. The disordered and globular protein regions′ fundamentally different structures are reflected in their melting diagrams, allowing one to directly determine the level of disorder in a specific protein structure. Intrinsically disordered proteins bind water more strongly than globular proteins, which is shown by the somewhat higher temperature values where mobile hydration water first appears but with a significantly higher heterogeneity in the energy distributions of protein–water interactions.

## Introduction

Globular and disordered proteins have fundamentally different characteristics that also manifest in their distinct physiological functions.^1^ The structural preference of a protein is encoded in its amino acid sequence, and the level of disorder in a specific protein can be measured on a continuous scale, with completely disordered and fully ordered structures on the two ends. Several experimental techniques exist that can determine the disorder tendency of a polypeptide chain, but few of them provide direct observations of their actual structural state. One such method is wide-line ^1^H NMR that directly measures the properties of the hydration shell of proteins, delivering exact, measurable values of their disorder/order content. In the present work, we perform a comparison between several globular (ordered) and disordered proteins to be able to get general features of the subzero mobile hydration of these two groups. We investigate the common properties of mobile hydration water on the basis of wide-line ^1^H NMR signal intensity measurements.

Proteins come in many different forms and shapes, as represented by thousands of different structures deposited in the Protein Data Bank.^2^ The two main structural categories that protein structures can take are globular and disordered, but there are several transient structural states that a particular protein can adopt under specific conditions.

Globular (ordered) proteins exist in an enormous variety of three-dimensional structures, which can be described by the coordinates of all its atoms. Nearly all of them contain substantial numbers of α-helices and β-sheets folded into a compact structure that is stabilized by both polar and nonpolar interactions. The globular three-dimensional (3D) structure forms spontaneously and is maintained as a result of interactions among the side chains of the amino acids. Most often, the hydrophobic amino acid side chains are buried, closely packed, in the interior of a globular protein and out of contact with water. Hydrophilic amino acid side chains lie on the surface of the globular proteins exposed to the water.

Wide-line ^1^H NMR measures the intensity of the proton signal, coming from all parts of the protein solution in question: the signal of ^1^H nuclei of the proteins and other molecules of the solvent water (both fluid and solid-ice). The signal intensity just after the generating radiofrequency pulse is in direct proportion to the number of the atomic nuclei concerned. The NMR signals of different materials can be distinguished upon their time-decay properties. In frozen solutions, only the water molecules interacting with proteins or other substances remain mobile below 0 °C, in a certain temperature range. The relative ratio of mobile water molecules, compared to the whole water content, can be measured as a function of temperature. It can be represented as a melting diagram (MD), which is the fraction of mobile protons (water molecules) (*n*), as a function of normalized functional temperature (*T*_fn_) or potential barrier (*E*_a_) belonging to protein–water interactions.^3^ The functional temperature is the temperature multiplied with the universal gas constant and then normalized to the melting point of bulk ice as *T*_fn_ = *T*/273.15 K to arrive at normalized functional temperature. The potential barrier is *T*_fn_ multiplied with the molar enthalpy of fusion for water (6.01 kJ mol^–1^)^4^.

The amount of the mobile hydration water and the shape of the MD at subzero temperatures together provide excellent means to distinguish globular proteins from disordered proteins. The shape of MD also gives information about the presence of secondary structural elements.

There are dynamic parameters introduced to determine the ratio of heterogeneity to homogeneity in the energy distribution of the proteins′ surface: (*HeR*),^3,5^ the ratio of the mobile water bound with heterogeneous energy distribution (*HeR*_n_),^5^ and the heterogeneity measure (*HeM*),^3,5^ which characterizes the extent of heterogeneity in the energy distribution of protein–water interactions close to 0 °C (see the Experimental Section).

MDs provide direct information about the behavior of differently bound water molecules and on the potentials determining the protein–water interaction. The MDs can be used as unbiased, direct measurements of the structures of hydration shells of proteins. Disordered and globular protein regions have fundamentally different structures, and these differences are expected to be reflected in their MDs, allowing us to directly determine the level of disorder in a specific protein structure. To test this hypothesis, we measured and compared the MDs of several globular and disordered proteins using wide-line ^1^H NMR.

In order to assess the structural tendencies of the proteins, we can use different in silico prediction algorithms. IUPred3^6^ identifies Intrinsically Disordered Protein Regions (IDPRs, i.e., regions that lack a stable monomeric structure under native conditions) using a biophysics-based model. IUPred3 returns a score between 0 and 1 for each residue, corresponding to the probability of the given residue being part of a disordered region. Importantly, IDPRs often harbor binding regions that are able to specifically interact with different molecular partners and such disordered binding regions are identified using the ANCHOR2 prediction algorithm.^7^ It also assigns to each residue a score between 0 and 1 (with higher values pertaining to higher interaction capacity), representing the probability of the given residue to be part of a disordered binding region.

As an overall feature of the globular/ordered proteins, the ones studied here (bovine serum albumin (BSA),^8^ bovine β-casein,^9^ ubiquitin (UBQ),^10^ lysozyme^11^) all show low IUPred scores, well below 0.5 (0.19(9) on average). Their ANCHOR scores are also very low (0.23(10) on average), indicating that they have no or very few disordered binding regions (for detailed data, see the Supporting Information and Figure 3).

Water around proteins can be divided into three main categories in general.^12^ (1) The bulk water surrounds the protein molecule with greater separation than the van der Waals connection. (2) The water molecules making hydrogen bonds with the charged or polar amino acids in the cavities inside the protein and that are bound individually.^13^ (3) Hydration water on the protein surface, directly interacting with the protein.^14,15^ Different types of protein–water interactions are characterized by different energies, resulting in different melting temperatures. Therefore, a constant region in the MD indicates a single type of interaction between mobile hydration water molecules and solvent-accessible surface areas (SASAs) of the protein.

**Figure 1:**
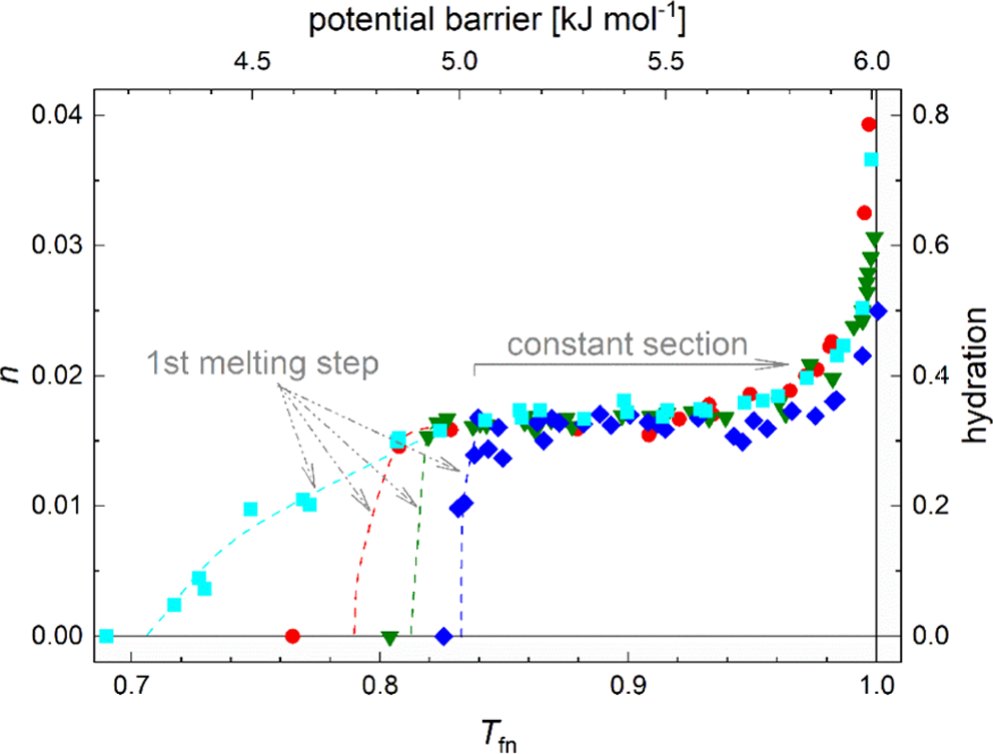
Melting diagrams of globular/ordered proteins: BSA (red circles), β-casein (green triangles), ubiquitin (dark blue diamonds), and lysozyme (light blue squares).

The first amount of mobile hydration water consists of a single type when the melting step is steep, see e.g., ubiquitin (Figure 1), which reflects no variety in the melting point. In other cases (e.g., lysozyme), the melting occurs continuously at the very beginning (at low temperatures), but after it reaches a certain value, the melting curve becomes constant, unchanging for a wide temperature range (Figure 1). There is continuous melting, therefore continuously increasing the amount of mobile hydration water in the vicinity of 0 °C. At these elevated temperatures, more and more different types of hydration water melt, and they become mobile, and the melting curve increases due to the increasing amount of mobile hydration water (Figure 1). The first amount of mobile water (see the first melting step in Figure 1) is bound most probably by H-bonds since this is a type of bond with discrete bonding distances and energies. Also, van der Waals type of interactions can be active at elevated temperatures or before the constant section of the MD with increasing mobile hydration water quantities.

The MDs of globular proteins are constant immediately after the first amount of mobile water appears and remain constant until −5 to −10 °C (Figure 1). *n* = 1 when the whole water amount is mobile, i.e., water is the liquid in the total volume of the sample.

**Figure 2:**
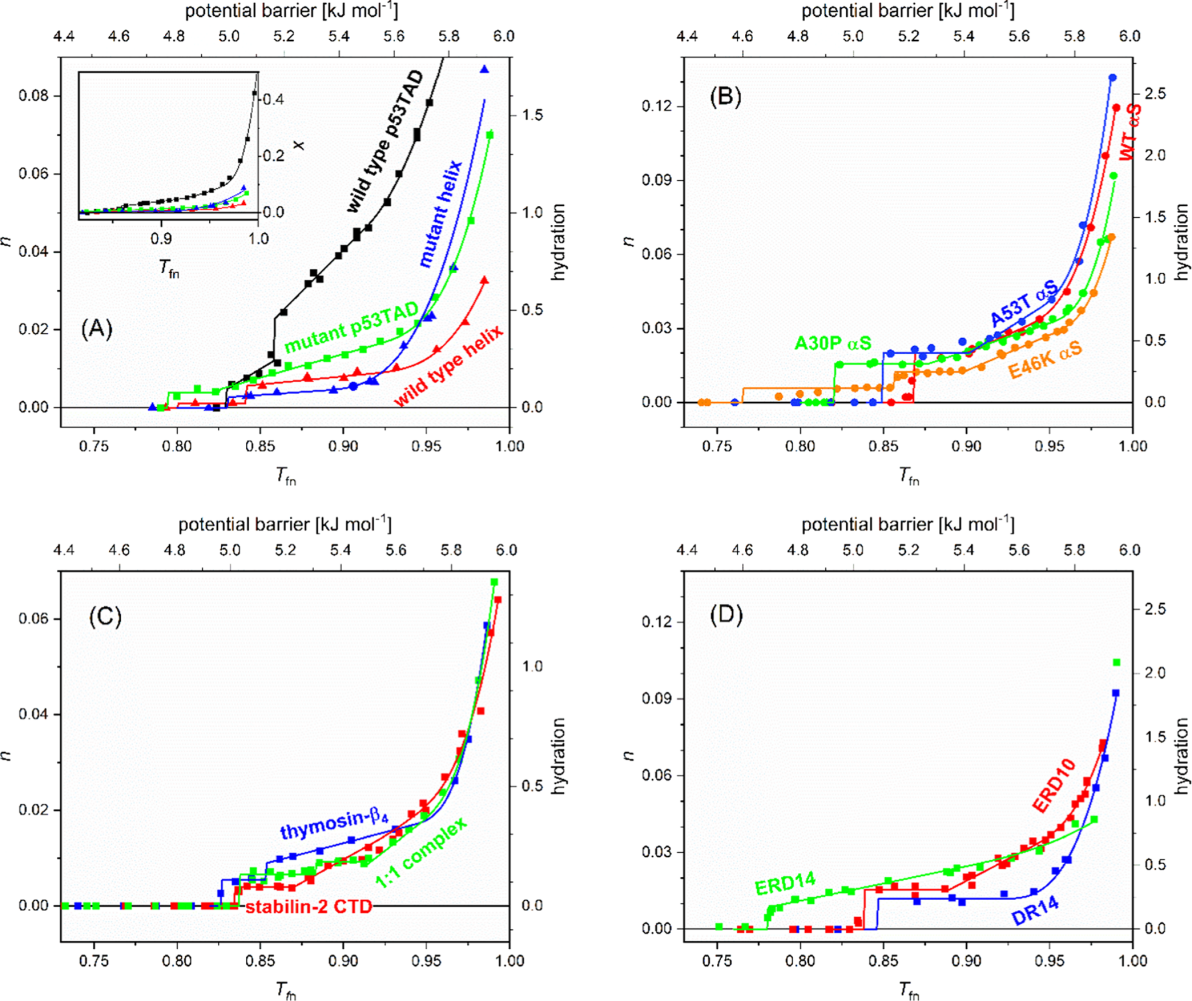
Melting diagrams of intrinsically disordered proteins. (A) wild-type p53 TAD (black), mutant p53 TAD (green), helical part of p53 TAD: wild-type (red) and mutant (blue); (B) α-synucleins: wild-type (red), A30P (green), E46K (orange), A53T (blue); (C) thymosin-β_4_ (blue), stabilin-2 CTD residues 2501–2551 (red), and their 1:1 complex (green); (D) Dr14 (blue), ERD10 (red), and ERD14 (green) proteins. The line is the analytical description (eq 1) applied.

**Figure 3:**
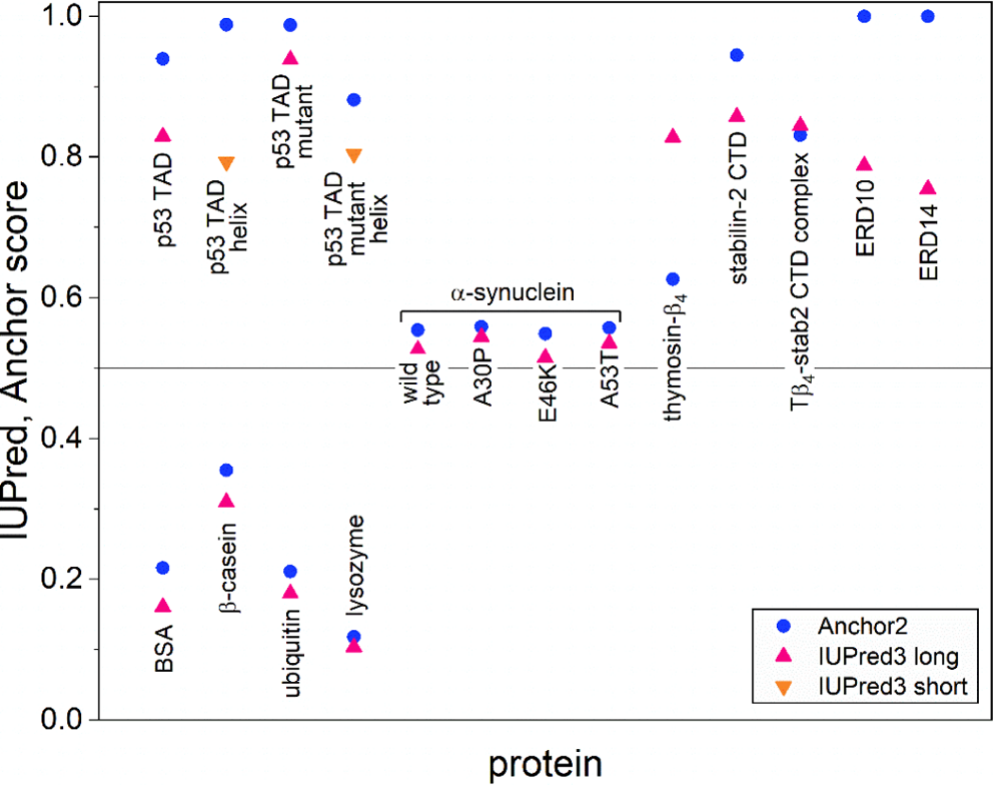
IUPred3 identifies Intrinsically Disordered Protein Regions (IDPRs) and returns a score between 0 and 1, corresponding to the probability of a disordered region. IDPRs often harbor binding regions identified by the ANCHOR2 prediction algorithm, which assigns a score representing the probability of a disordered binding region.

The structure of intrinsically disordered proteins (IDPs) cannot be described with a single defining structure because an IDP by definition can be characterized by a coexisting multitude of conformations, called a structural ensemble. IDPs show completely different MDs (Figure 2) than the ordered or globular ones. They have MDs, which show continuous increments in the whole temperature range with mobile hydration water molecules. Only a narrow range of a constant amount of mobile hydration water can be an exception from this at the lowest temperatures if the given protein contains some parts of relatively stable secondary structural elements. The secondary elements can generate the constant melting diagram section. The studied disordered proteins have high (i.e., above 0.5) IUPred3 and ANCHOR2 values (0.7(1) IUPred3, 0.8(2) ANCHOR2 scores on average, Supporting Information and Figure 3).

## Experimental Section

### Proteins

The following lyophilized proteins were purchased from Sigma-Aldrich: Bovine β-casein (Cat. No. C6905), ubiquitin (UBQ) (Cat. No. U6253), hen’s egg white lysozyme (Cat. No. 62971), and bovine serum albumin (Cat. No. A2153).

#### Expression and Purification of Proteins

##### p53 TAD and Its Variant^16,17^

The full-length p53 TAD (1–73) was expressed and purified as described previously.^16^ The p53 helix peptide (15–29) and its mutant variant with helix-breaking mutations (19F → A, 22L → Ala, 23W → A, and 25L → A) were synthesized by a solid phase method with Multiple Peptide Synthesizer APEX 348Ω (Advanced Chemtech). The sequences of the synthesized wild-type and mutant p53 TAD peptides are as follows:

WT: SQETFSDLWKLLPEN-NH_2_

Mutant: SQETASDAAKLAPEN-NH_2_

The C-termini of all of the synthesized peptides were amidated. The peptides were purified by high-performance liquid chromatography (HPLC) using Vydac C18 columns, and their masses were confirmed by matrix-assisted laser desorption ionization-time of flight (MALDI-TOF) mass spectrometry.

##### α-Synuclein and Disease-Related Mutants: A30P, E46K, A53T^18,19^

Expression and purification of recombinant human wild-type and mutant α-synuclein variants in a pRK-172-based expression system was performed as described.^20^ Briefly, expression of the proteins was performed in *Escherichia coli* Bl21(DE3) in a pT7-7-based expression system after IPTG induction. Bacterial cell pellets were harvested by centrifugation and resuspended in 10 mM Tris-HCl, pH 8.0, 1 mM EDTA, and 1 mM cOmplete protease inhibitor cocktail. After cell lysis, streptomycin sulfate-precipitated DNA was removed by centrifugation, and an ammonium sulfate precipitation step was performed. After centrifugation, the pellet was dissolved in 10 mM Tris-HCl, pH 7.4, and 1 mM cOmplete, and loaded onto a Resource Q anion exchange column on an AKTA Explorer chromatography system (GE Healthcare). The purity and integrity of the purified proteins were confirmed by sodium dodecyl sulfate polyacrylamide gel electrophoresis (SDS-PAGE). Peak fractions were collected and dialyzed against double-distilled water before lyophilization.

##### Thymosin-β_4_, Stabilin-2 Cytoplasmic Domain, and Their Complex^21,22^

DNA encoding human Tβ_4_ (UniProt: P62328) was synthesized by MWG Biotech AG and cloned into a pET22b cloning vector. A stop codon was added to ensure that the protein was expressed without a His-tag. After overnight induction, cells were pelleted and lysed by sonication. Cell debris was removed by centrifugation and the supernatant was boiled in a water bath for 5 min and centrifuged. 3% Perchloric acid was added to the supernatant and precipitated proteins were removed by centrifugation. The pH of the supernatant was adjusted to 7.0 by adding 5 M KOH. The final protein solution was purified on a reversed phase (RPC) column (GE Healthcare, 17-1182-01), on an AKTA Explorer system (GE Healthcare). Eluted Tß_4_ was dialyzed with distilled water and lyophilized.

The DNA coding for the truncated intracellular region (amino acids 2501–2551) of human stabilin-2 (UniProt: Q8WWQ8) was synthesized by the MWG Biotech AG company and then cloned into a pETARA cloning vector for the expression of the GST-tagged protein construct. Protein expression was induced overnight. After sonication in lysis buffer, the cell extract was centrifuged, and the supernatant was loaded on a GSTrap FF column. After elution, an overnight TEV protease treatment was used to remove the GST-tag. The mixture was boiled in a water bath for 5 min and centrifuged. The purified protein was dialyzed against 20 mM ammonium acetate (pH = 6.8) and lyophilized.

Complex formation was achieved by mixing equimolar amounts of the two proteins in distilled water and lyophilizing the mixture.

##### Early Response to Dehydration 10 (ERD10) and 14 (ERD14)^23,24^

The expression of the recombinant proteins (ERD10 and ERD14) was performed as described in refs (23,24). Briefly, the induced *E. coli* cells were harvested by centrifugation and were disrupted by sonication in the lysis buffer. After centrifugation, the supernatant was placed in a boiling-water bath for 5 min for heat fractionation, and aggregated proteins were removed by centrifugation. The supernatant was purified on a DEAE cellulose (Pharmacia) column using an Akta Explorer System (GE Healthcare). The protein fractions detected by SDS-PAGE were pooled and dialyzed into 100 mM acetic acid and 1 mM EDTA, pH 6. The protein sample was loaded onto an equilibrated CM-Sepharose column (Pharmacia) where ERD10 and ERD14 appeared in the flow through. The protein fractions were dialyzed into Millipore water for lyophilization.

In the sample preparation, the mass of the lyophilized proteins (without any further refinement) was measured, and an appropriate amount of water was added to obtain the nominal concentrations, 50 mg/mL with double-distilled water used as a solvent.

### Wide-Line NMR

The aqueous protein solutions were prepared by dissolving the appropriate amount of the actual protein in double-distilled water. Detailed descriptions of the applied NMR methods were reported earlier.^25,26^ Briefly, the intensity of the NMR signal is measured as the amplitude of free induction decay (FID) signal, extrapolated to *t* = 0 (start of the pulse). The extrapolated amplitude is proportional to the number of contributing nuclei. The measurements were done on cooled and reheated samples, in the temperature range of −70 to +30 °C. All of the protons were in a mobile state (water molecules in liquid state) above 0 °C, and the NMR intensities were normalized to this value. The effect of freezing on the protein solutions was controlled by the comparison of NMR parameters obtained before and after a freeze–thaw cycle, at temperatures above 0 °C. The freeze–thaw cycle caused no observable changes (cold denaturation) for the studied protein solutions. Temperature was controlled by an open-cycle Janis cryostat with an uncertainty better than ±1 K. ^1^H NMR measurements and data acquisition were accomplished by a Bruker AVANCE III NMR spectrometer at a frequency of 82.4 MHz, with a stability better than that of ±10^–6^. The data points in the figures are based on spectra recorded by averaging signals to reach a signal-to-noise ratio better than 50. We varied the number of averaged NMR signals to achieve the desired signal quality for each sample and unfrozen water. The extrapolation of the FIDs to zero time was done by fitting a sum of Gaussians.

Dimensionless (normalized) physical quantities are applied as fundamental temperature on the abscissa, which is an energy/temperature scale defined in energy units, and moreover as the fraction of mobile protons (water molecules), *n* on the ordinate. In this scale, by definition, *T*_f_ = *RT*. One can relate the melting temperature(s) of the interface or particular points of MD to the melting point of bulk ice (water), thus introducing the *T*_fn_ normalized fundamental temperature as *T*_fn_ = *RT*/(*R*·273.15 K) = *T*/273.15 K. On the *T*_fn_ scale, the melting point of bulk ice (water) is 1, whereas that of the interfacial ice (water) is a positive number that falls between 0 and 1. The molar heat of fusion of water is the thermal activation energy required to overcome the potential energy barrier that hinders the movement of water molecules in bulk ice. We relate the rotational energy barriers in the interface to those in bulk water.

## Results and Discussion

To obtain the necessary information about the hydration states of a protein, MD can be described analytically and fitted with a power series
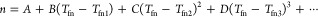
1

The lowest temperature at which mobile water molecules can be detected is denoted by *T*_fn0_. This quantity is not a fitting parameter, but it is measured as the first temperature where *n* > 0. Fitting of the higher-temperature regions in the melting diagram is performed by sections with increasing temperature limits, i.e., the fitting procedure starts with the lowest temperature range. The temperature value dividing the first two sections, namely, constant and linearly increasing MD trends, is given by the parameter *T*_fn1_. Likewise, the next two sections are divided by the trend change from linear to quadratic at *T*_fn2_ and finally the trend becomes cubic at *T*_fn3_. At the constant mobile hydration water amount and constant *n* region, if any, the value of *A* is determined and kept for further analysis. Then, parameters *B* and *T*_fn1_ can be fitted at the linear section and further used in sections with higher powers. The section increasing quadratically is fitted to obtain the parameters *C* and *T*_fn2_. A cubic section can occur at temperatures just below 0 °C, which is described by parameters *D* and *T*_fn3_.

**Figure 4:**
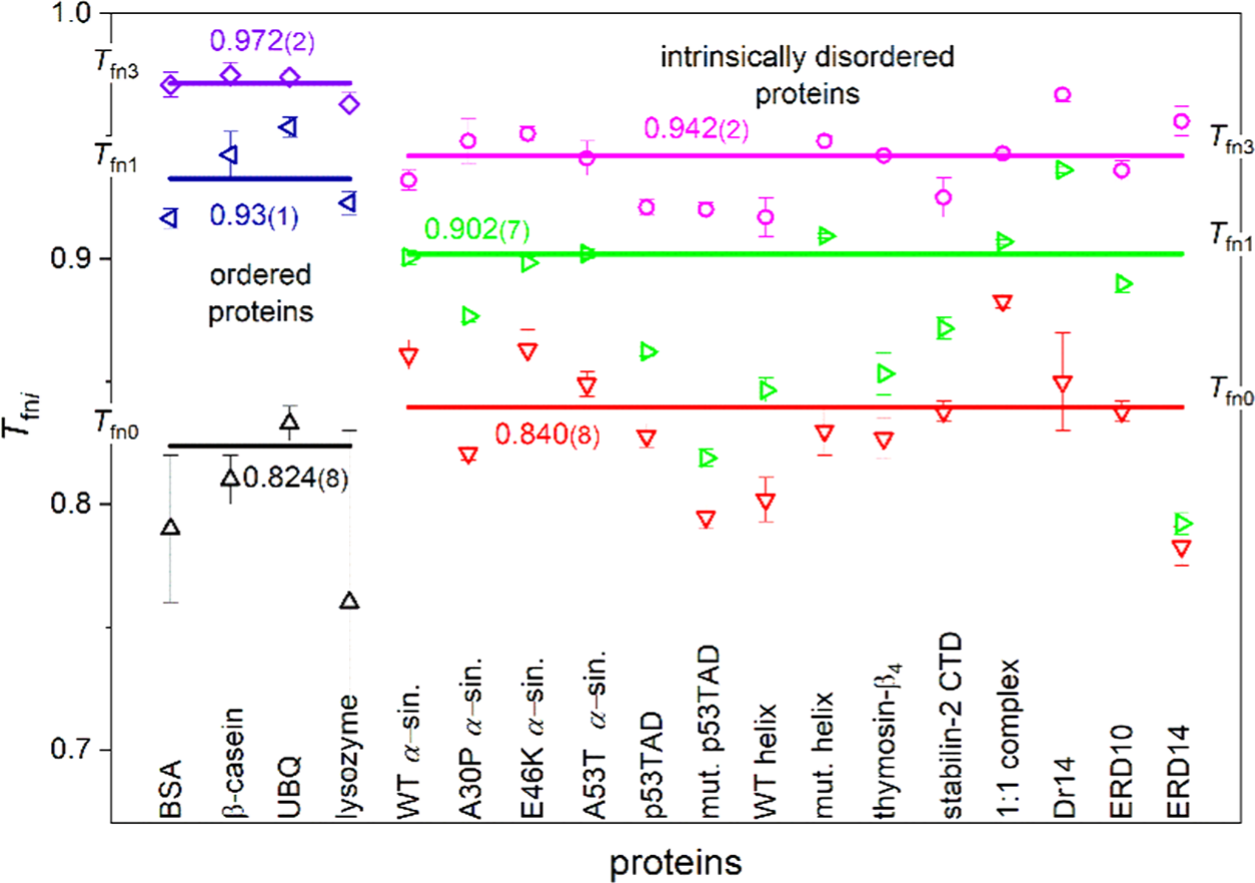
Trend changing points of the melting diagrams (MDs) according to the type of protein order. *T*_fn1_ values denote the MD point where linear elevation starts after a constant section. Similarly, the cubic MD section starts at *T*_fn3_. The lines represent error-weighted averages with values marked at the lines.

**Figure 5:**
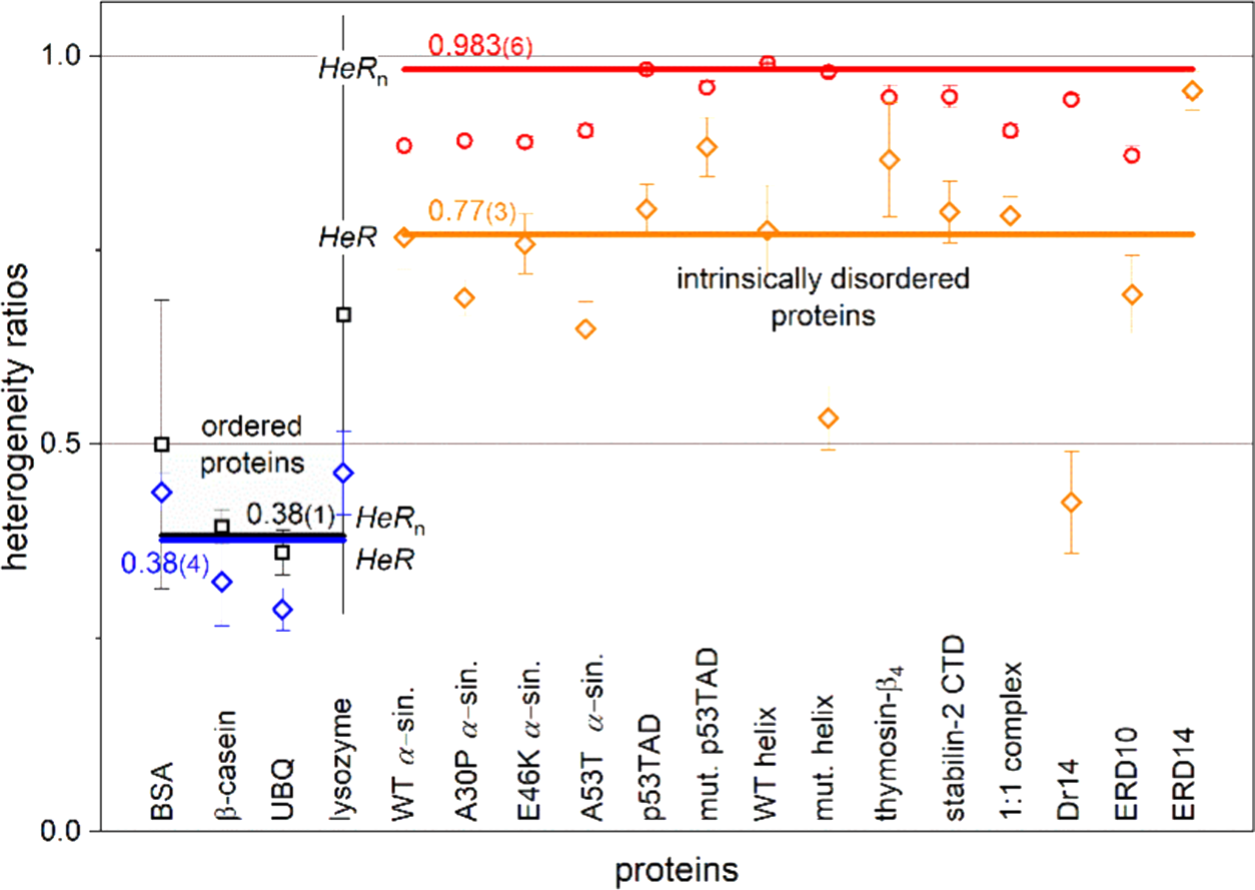
Dynamic parameters *HeR* (ratio of homogeneity) and *HeR*_n_ (ratio of the amount of heterogeneously bound water to the total number of bound water) for ordered and disordered proteins. The lines represent error-weighted averages with values marked at the lines.

**Figure 6:**
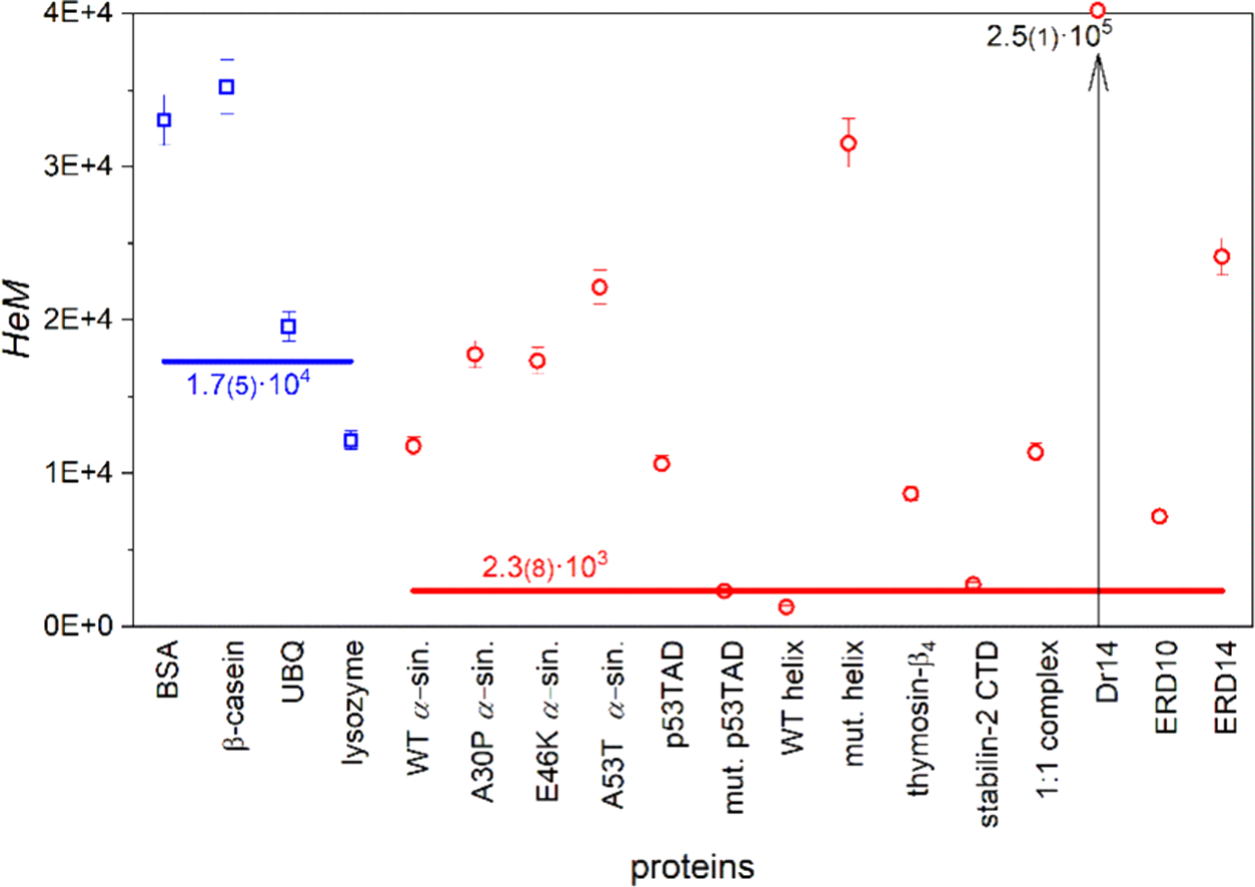
Dynamic parameter *HeM* (measure of heterogeneity) for ordered (blue squares) and disordered (red circles) proteins. The lines represent error-weighted averages with values marked at the lines.

The following derived quantities are used for general characterization of the proteins:^3,5^ (i) the ratio of heterogeneity to homogeneity, *HeR* = (1 – *T*_fn1_)/(1 – *T*_fn0_),^3^ (ii) the ratio of the amount of heterogeneously bound water (in terms of energy distribution) and the total number of bound water (homogeneously bound, *n*_ho_, plus heterogeneously bound, *n*_he_), *HeR*_n_ = *n*_he_/(*n*_ho_ + *n*_he_)^5^, and (iii) the measure of heterogeneity, *HeM* = (*B* + 2*C* + 3*D*)/(1 – *T*_fn1_)^3^. The actual values of these quantities for the different ordered and intrinsically disordered proteins are presented in Figures 4–6.

In more detail, *HeR* informs about the extent of the disordered or ordered nature of a protein by comparing the temperature distances from 0 °C of mobile water’s occurrence and the linear trend of MD. *HeM* gives information about the intensity of *n*’s growth close to 0 °C, which can be interpreted as a characteristic of the heterogeneity of the protein–water interactions in this zone.

Detailed analyses of the critical *T*_fn0_, *T*_fn1_, and *T*_fn3_ points markedly reflect the differences between the ordered and (intrinsically) disordered proteins. The *T*_fn0_ values (Figure 4) show where the constant section starts in the MD. They are narrowly equal within an error for the two types of proteins, with the ordered proteins having a slightly lower value than the disordered ones (Table S3). Here, there are already mobile hydration water molecules that are most probably attached to solvent-accessible surface areas (SASAs) determined by the second-order protein structures. The amount of the hydration water molecules becoming mobile at *T*_fn0_ is , while , meaning that ordered proteins bind one and a half times as much water as disordered ones in their constant MD section just before the (linearly) increasing section. The energy threshold, *T*_fn0_, is a limiting characteristic for this type of water molecules bound to the SASAs of the protein, determined by secondary structures, characterized with protein–water interaction energies less than or equal to *E*_a,0_ = *T*_fn0_ × 6.01 kJ mol^–1^ (normalized functional temperature multiplied with the molar heat of fusion for water^4^). For the proteins where this energy threshold appears as a step in the MD, this means a single activation energy for the rotation of the hydration water molecules becoming mobile here. This single activation energy is characteristic of hydrogen bonds. In other cases, the energy threshold corresponding to *T*_fn0_ is preceded by a linearly changing section of the MD. This behavior refers to a continuity of water–protein interaction energies, which is characteristic of van der Waals forces. Based on the previously described differences in their MDs, we can state that initially, the ordered proteins are surrounded by substantially more water than the IDPs. This difference is then even overcompensated by the IDPs as their MDs are constantly increasing after the short constant sections in contrast to the ordered proteins that have constant MDs almost in the whole subzero range. Another important difference between the two structural protein classes is that the ordered proteins have hydration water with one single type of protein–water bonds, which is an energetically fully homogeneously bound hydration layer up to nearly the melting point of bulk water. In contrast, the IDPs have continuously growing mobile hydration with energetically heterogeneously bound water molecules from *T*_fn1_ as both the bond types as the bonding energies change with temperature/potential barrier of the motion of hydration water.

The *T*_fn1_ values are higher (closer to 0 °C) for the ordered proteins than for the IDPs, and consequently, the same is true for the *T*_fn1_ – *T*_fn0_ distances. The distance (*T*_fn1_ – *T*_fn0_ = 0.11(2)) is almost twice greater for the ordered proteins (Figure 4 and Table 1) than the same value for the IDPs (*T*_fn1_ – *T*_fn0_ = 0.06(1)). This is because the ordered proteins′ MDs are determined by the wide constant section so the increase of the amount of mobile hydration water starts only at greater potential energy barrier values. They have SASAs mostly determined by hydrophobic amino acid residues of protein–water interactions with a homogeneous energy distribution. These residues belong mainly to secondary structures such as α-helices and β-sheets. The narrower sections of MDs with increasing trends are 1 – *T*_fn1_ = 0.07(1) wide for ordered proteins in contrast to IDPs with a width of 1 – *T*_fn1_ = 0.098(6). More water molecules start to move at *T*_fn1_. After the constant *n*(*T*_fn_) section (Figure 4), an elevation follows until the melting point of water at *T*_fn_ = 1. This means constantly entering newer mobile water molecules, and consequently, the MD has here at *T*_fn1_ ≤ *T*_fn_ ≤ 1, a nonzero slope, with the new mobile water molecules having higher and higher activation energies. The linear growth of the amount of mobile hydration water ends at *T*_fn2_, where a quadratically increasing MD section starts, or at *T*_fn3_, if the quadratic section is lacking, like in the cases of the studied proteins. The section of more rapid cubic growth starts at *T*_fn3_ and lasts until *T*_fn_ = 1. The entrant new mobile hydration water molecules are bound stronger to the proteins (the potential barrier affecting their motion is higher). They hydrate strongly hydrophilic or ionic residues.

**Table 1:** Critical Points *T*_fn0_, *T*_fn1_, and *T*_fn3_ of the MDs and Dynamic Parameters *HeR*, *HeR*_n_, and *HeM* for Ordered and Disordered Proteins^a^

	type of protein	
	ordered	disordered
*T*_fn0_	0.824(8)	0.840(8)
*T*_fn1_	0.93(1)	0.902(7)
*T*_fn3_	0.972(2)	0.942(2)
*HeR*	0.38(4)	0.77(3)
*HeR_n_*	0.38(1)	0.983(6)
*HeM*	1.7(5) × 10^4^	2.3(8) × 10^3^

a Error-weighted average values. The error in the last digit is given in parentheses.

*HeR* is twice as large for the IDPs than the ordered proteins (Table S3 and Figure 5). It is a consequence of the fact that the *T*_fn1_ values are close to 1 for the ordered proteins, while the *T*_fn1_ values of the IDPs are close to the *T*_fn0_ values. Accordingly, the IDPs contain extensive sections with heterogeneous protein–water interaction centers. The difference between the two types of proteins is even greater in the *HeR*_n_ parameters (Table S3 and Figure 5). This means that in the IDPs of *HeR*_n_ = 0.983(6), almost all water molecules are heterogeneously bound (different bond types and energies) as *n*_he_ = 0.14(1) and *n*_ho_ = 0.0099(5). On the other hand, ordered proteins are quite homogeneous in their protein–water bonds, and 66% of them are of the same type as the width of the constant MD section shows. The amount of the homogeneously bound water, *n*_ho_ = 0.0165(2), is practically the same as the amount of the heterogeneously bound water, *n*_he_ = 0.017(1). Although the *HeM* parameters (Figure 6) have a wide distribution for both protein types, the average values still show clear separation. The *HeM* averages greater by one order of magnitude refer to more intensively increasing mobile hydration water amounts near 0 °C for ordered proteins than in the case of IDPs (Table S3).

To picture dynamic contributions to IDP function requires information about properties of hydration water and its interactions with IDPs, which wide-line ^1^H NMR can provide. We have demonstrated by wide-line ^1^H NMR measurements already in 2005 that IDPs bind a significantly larger amount of water than globular proteins and also analyzed relaxation times.^26^ In summary, our present results suggest that IDPs bind water more strongly than globular proteins, which is shown by not only the somewhat higher *T*_fn0_ values but with a significantly higher heterogeneity in the energy distributions of the protein–water interactions. These observations are in line with recently published results based on megahertz-to-terahertz dielectric spectroscopy and molecular dynamics simulations,^27^ where the dynamics of the water molecules surrounding IDPs was found to be exceeding that of the globular proteins. These observations can be directly related to the differences in the amino acid compositions between the two protein classes and the alterations in their solvent-accessible surfaces. IDPs are highly enriched in hydrophilic residues, which strongly interact with water, while the surfaces of globular proteins are generally dominated by secondary structural elements, resulting in a less tightly bound, larger hydration shell.

## Supporting Information Available

Lengths in numbers of amino acids, molecular weights, and IUPred and ANCHOR scores for globular proteins (Table S1); lengths in numbers of amino acids, molecular weights, and IUPred and ANCHOR scores for disordered proteins (Table S2); and parameter values from fitting eq 1 to the measured relative ratio of mobile water molecules compared to the whole water content vs functional normalized temperature, *n* vs *T*_fn_ (*T*_fn_ = *T*/273.15 K) (Table S3) (PDF)
