# Supplementary material for: General Characterization of Properties of Ordered and Disordered Proteins by Wide-Line 1H NMR
Source: ACS Omega. 2024 May 22;9(22):23468–75. doi: 10.1021/acsomega.4c00517 (PMC11154930; doi:10.1021/acsomega.4c00517)
Supplement: Supplementary file 1 — ao4c00517_si_001.pdf [file ao4c00517_si_001.pdf]

General characterization of properties of ordered and disordered proteins by wide-line ^{1}H NMR

Mónika Bokor[*1], *Ágnes Tantos*[2]

[1] Institute for Solid State Physics and Optics, HUN-REN Wigner Research Centre for Physics, 1121 Budapest, Hungary

[2] Institute of Enzymology, HUN-REN Research Centre for Natural Sciences, 1117 Budapest, Hungary

IUPred3[1] and ANCHOR2[2] predictions are presented in Tables 1 and 2 together with a brief description of the molecular function of the proteins investigated. IUPred3 identifies Intrinsically Disordered Protein Regions (IDPRs, i.e. regions that lack a stable structure under native conditions) using a biophysics-based model. IUPred3 returns a score between 0 and 1 for each residue, corresponding to the probability of the given residue being part of a disordered region. A binding region of an IDRP which is able to specifically interact with a globular domain. Binding regions are identified using the ANCHOR2 prediction algorithm. It assigns to each residue a score between 0 and 1, representing the probability of the given residue to be part of a disordered binding region.

Table S1. Basic properties of the studied globular proteins (low IUPred scores).

Name	aa length	MW	Average IUPred, ANCHOR score	Molecular function
**bovine serum albumin (BSA)[3]**	607	69,293 Da	0.1608, 0.216	Binds water, Ca^{2+}, Na^{+}, K^{+}, fatty acids, hormones, bilirubin and drugs. Its main function is the regulation of the colloidal osmotic pressure of blood. Major Zn transporter in plasma, typically binds about 80% of all plasma Zn.
**bovine β-casein[4]**	226	5,382 Da	0.3100, 0.355	Important role in determination of the surface properties of the casein micelles.
**ubiquitin (UBQ)[5]**	76	8,565 Da	0.1802, 0.211	Tagging and tracking proteins, causing their elimination. Ubiquitination also occurs in most forms of protein degradation.
**lysozyme[6]**	148	16,537 Da	0.1038, 0.118	Primarily a bacteriolytic function; lysozymes in tissues and body fluids are associated with the monocyte-macrophage system and enhance the activity of immunoagents.

Table S2. Basic properties of the studied disordered proteins (high IUPred and ANCHOR scores).

Name	aa length	MW	Average IUPred, ANCHOR score	Molecular function
**p53 transcriptional activation domain (TAD)**[7]	73	8,145 Da	0.8295, 0.940	Potent transcriptional activator, regulates cell growth negatively. The peptide lacks any regular secondary structure in solution. It has domains that participate in sequence-specific DNA biding, tetramerization, and transcriptional activation.
**helix motif of p53 TAD (15-29)**[8]	15	1,807 Da	0.7928, 0.988	It is a peptide with a helix pre-structuring property.
**mutant p53 TAD (F19A, L22A, W23A, L25A)**[8]	73	7,870 Da	0.9382, 0.987	It is a mutant protein, without helix-forming propensity.
**mutant helix motif (15-29; F19A, L22A, W23A, L25A)**[8]	15	1,532 Da	0.8042, 0.820	It is a mutant peptide with a disabled helix-forming propensity.
**wild type α-synuclein**[9]	140	14,460 Da	0.5261, 0.554	It is highly expressed and conserved, found in the presynaptic terminals of neurons. Aggregated forms in the brain are thought to originate from the disequilibrium between the generation and clearance of *α*-synuclein monomers, leading to their accumulation of oligomers, protofibrils, and, ultimately, amyloid fibrils, which are considered pathological hallmarks of Parkinson's disease.
**α-synuclein A30P**[10]	140	14,486 Da	0.5445, 0.554	A30P, E46K, and A53T point mutants are associated with the early onset of familial Parkinson's disease. They may form functionally different molecular intermediates
**α-synuclein E46K**[10]	140	14,460 Da	0.5151, 0.549	

**α-synuclein A53T**[9]	140	14,490 Da	0.5356, 0.557	that alter the dynamics of protein aggregation.
**thymosin-β_4**[11]	43	4,921 Da	0.8283, 0.626	It has moonlighting functions. It can sequester actin, and it has important role in the regulation of the formation and modulation of the actin cytoskeleton. The multiple functions of $T\beta_4$ binds often weakly to its physiological partners, and forms structurally heterogeneous complexes with them, but it can fold upon binding. In the complexes, $T\beta_4$ engages in multiple fuzzy interactions.
**stabilin-2 CTD (2501-2551)**[11]	51	5,631 Da	0.8573, 0.944	It is an endocytic receptor for hyaluronic acid, binds $T\beta_4$. The complex of $T\beta_4$ with stabilin-2 is involved in the phagocytosis of apoptotic cells and $T\beta_4$ plays a role there.
**thymosin-β4—stabilin-2 CTD complex**[11, 12]	94	10,534 Da	0.8447, 0.831	The thermal trends of MDs showed that isolated $T\beta_4$ and stabilin CTD, as well as their complex, are intrinsically disordered proteins. Weak binding was confirmed between $T\beta_4$ and stabilin CTD. It was suggested that the proteins become slightly more disordered in the complex.
**ERD10**[13]	260	29,547 Da	0.7878, 1.00	These proteins (for early response to dehydration) are disordered plant chaperones and play key roles in helping plants survive in harsh conditions, and they are indispensable for seeds to remain viable. They are members of the dehydrin family that accumulate in response to abiotic environmental stresses, such as high salinity, drought, and low temperature, in Arabidopsis (Arabidopsis thaliana). They are intrinsically disordered proteins and have chaperone activity of rather wide substrate specificity.
**ERD14**[14]	185	20,786 Da	0.7548, 1.00	

Table S3. Parameter values from fitting Equation 1 to measured relative ratio of mobile water molecules, compared to the whole water content *vs.* functional normalized temperature, *n vs.* T_{fn} ($T_{fn} = T$ / 273.15K)

**Name**	$\boldsymbol{T_{fn0}}$	$n_{ho} = A$	*B*	$\boldsymbol{T_{fn1}}$	*D*	$\boldsymbol{T_{fn3}}$	*n*(1)	$n_{he} = n(1) - n_{ho}$	HeR_n	*HeR*	*HeM*
**bovine serum albumin (BSA)[3]**	0.808(1)	0.0163(2)	0.064(7)	0.916(4)	$9(4)\cdot10^2$	0.971(5)	0.04(1)	0.028(1)	0.6(1)	0.44(2)	$3.3(2)\cdot10^4$
**bovine β-casein[4]**	0.821(2)	0.01665(9)	0.07(3)	0.94(1)	$7(4)\cdot10^2$	0.975(5)	0.031(1)	0.015(1)	0.47(1)	0.32(6)	$3.5(2)\cdot10^4$
**ubiquitin (UBQ)[5]**	0.838(1)	0.0160(2)	0.07(1)	0.954(4)	$3(1)\cdot10^2$	0.974(3)	0.025(1)	0.009(2)	0.35(1)	0.29(3)	$2.0(1)\cdot10^4$
**lysozyme[6]**	0.833(9)	0.0172(2)	0.040(7)	0.923(5)	$3(1)\cdot10^2$	0.963(5)	0.036(2)	0.0185(9)	0.5(1)	0.46(5)	$1.22(6)\cdot10^4$
**p53 transcriptional activation domain (TAD)[7]**	0.828(5)	0.009(1)	0.43(2)	0.862(2)	$4.9(8)\cdot10^2$	0.921(3)	0.31(3)	0.30(1)	0.971(5)	0.80(3)	$1.06(5)\cdot10^4$
**helix motif of p53 TAD (15-29)[8]**	0.802(9)	0.00110(4)	0.04(3)	0.847(5)	$7(2)\cdot10^1$	0.917(8)	0.045(6)	0.044(2)	0.976(2)	0.78(6)	$1.28(6)\cdot10^3$
**mutant p53 TAD (F19A, L22A, W23A, L25A)[8]**	0.795(5)	0.0040(6)	0.123(6)	0.819(3)	$1.4(2)\cdot10^2$	0.920(3)	0.099(5)	0.095(5)	0.959(8)	0.88(4)	$2.3(1)\cdot10^3$
**mutant helix motif**	0.83(1)	0.0037(8)	0.46(1)	0.9094(9)	$1.0(1)\cdot10^3$	0.948(2)	0.180(9)	0.176(9)	0.979(5)	0.53(4)	$3.2(2)\cdot10^4$

**(15-29; F19A, L22A, W23A, L25A)**[8]											
**wild type α-synuclein**[9]	0.870(3)	0.0202(6)	0.30(3)	0.901(3)	$4(1)\cdot10^2$	0.932(4)	0.175(9)	0.155(8)	0.884(8)	0.774	$1.18(6)\cdot10^4$
**α-synuclein A30P**[10]	0.821(3)	0.0157(2)	0.23(1)	0.877(2)	$7(5)\cdot10^2$	0.948(9)	0.143(7)	0.128(6)	0.890(6)	0.69(2)	$1.78(9)\cdot10^4$
**α-synuclein E46K**[10]	0.886(4)	0.0125(2)	0.29(2)	0898(2)	$6(1)\cdot10^2$	0.951(3)	0.113(6)	0.100(5)	0.889(7)	0.76(4)	1.73(9)
**α-synuclein A53T**[9]	0.849(5)	0.0202(6)	0.43(3)	0.902(2)	$7(3)\cdot10^2$	0.941(7)	0.21(1)	0.19(1)	0.904(7)	0.65(4)	$2.2(1)\cdot10^4$
**thymosin-β_4**[11]	0.831(4)	0.0055(4)	0.091(4)	0.853(9)	$4.2(4)\cdot10^2$	0.942(1)	0.10(2)	0.097(5)	0.95(1)	0.87(7)	$8.7(4)\cdot10^3$
**stabilin-2 CTD (2501-2551)**[11]	0.840(2)	0.00401(7)	0.19(2)	0.872(4)	$1.2(4)\cdot10^2$	0.925(8)	0.08(2)	0.073(4)	0.9(2)	0.8(4)	$2.7(1)\cdot10^3$
**thymosin-β4—stabilin-2 CTD complex**[11, 12]	0.883(3)	0.0075(4)	0.269(8)	0.9070(8)	$3.5(2)\cdot10^2$	0.943(1)	0.097(5)	0.090(4)	0.923(7)	0.79(2)	$1.14(6)\cdot10^4$
**ERD10**[13]	0.841(6)	0.0155(7)	0.34(3)	0.890(4)	$2.6(7)\cdot10^2$	0.936(4)	0.121(6)	0.106(5)	0.87(1)	0.69(5)	$7.2(4)\cdot10^3$
**ERD14**[14]	0.7825(7)	0.0081(1)	0.133(5)	0.792(5)	$1.7(8)\cdot10^3$	0.956(6)	0.178(9)	0.170(8)	0.954(3)	096(2)	$2.4(1)\cdot10^4$

References

1 Erdős, G.; Pajkos, M.; Dosztányi, Z. IUPred3: prediction of protein disorder enhanced with unambiguous experimental annotation and visualization of evolutionary conservation. *Nucleic Acids Res.* **2021**, *49(W1)*, W297–W303. DOI: 10.1093/nar/gkab408

2 Erdős, G., Dosztányi, Z. Analyzing Protein Disorder with IUPred2A. *Current Protocols in Bioinformatics*, **2020**, *70*, e99. DOI: 10.1002/cpbi.99

3 *Uniprot P02769*, https://www.uniprot.org/uniprotkb/P02769/entry

4 *Uniprot P05814*, https://www.uniprot.org/uniprotkb/P05814/entry

5 Camara–Artigas, A.; Plaza-Garrido, M.; Martinez–Rodriguez, S.; Bacarizo, J. New crystal form of human ubiquitin in the presence of magnesium. *Acta Crystallogr. F*, **2016**, *72*, 29–35. DOI: 10.1107/S2053230X15023390

6 *Uniprot P61626*, https://www.uniprot.org/uniprotkb/P61626/entry

7 Tompa, P.; Han, K.–H.; Bokor, M.; Kamasa, P.; Tantos, Á.; Fritz, B.; Kim, D. H.; Lee, C.; Verebélyi, T.; Tompa, K. Wide-line NMR and DSC studies on intrinsically disordered p53 transactivation domain and its helically pre-structured segment. *BMB Rep.* **2016**, *49*, 497–501. DOI: 10.5483/bmbrep.2016.49.9.037

8 Tompa, P.; Han, K.–H.; Bokor, M.; Kamasa, P.; Tantos, Á.; Fritz, B.; Kim, D. H.; Lee, C.; Verebélyi, T.; Tompa, K. Wide-line NMR and DSC studies on intrinsically disordered p53 transactivation domain and its helically pre-structured segment. *BMB Rep.* **2016**, *49*, 497–501. DOI: 10.5483/bmbrep.2016.49.9.037

9 Házy, E.; Bokor, M.; Kalmár, L.; Gelencsér, A.; Kamasa, P.; Han, K.–H.; Tompa, K.; Tompa, P. Distinct hydration properties of wild-type and familial point mutant A53T of a-

synuclein associated with Parkinson's disease. *Biophys. J.* **2011**, *101*, 2260–2266. DOI: 10.1016/j.bpj.2011.08.052

10 Bokor, M.; Házy, E.; Tantos, Á. Wide-line NMR melting diagrams, their thermodynamic interpretation, and secondary structure predictions for A30P and E46K α-synuclein. *ACS Omega*, **2022**, *7*, 18323–18330. DOI: 10.1021/acsomega.2c00477

11 Tantos, Á.; Szabó, B.; Láng, A.; Varga, Z.; Tsylonok, M.; Bokor, M.; Verebélyi, T.; Kamasa, P.; Tompa, K.; Perczel, A.; Buday, L.; Lee, S. H.; Choo, Y.; Han, K.–H.; Tompa, P. Multiple fuzzy interactions in the moonlighting function of thymosin-β_4. *Intrinsically Disordered Proteins*, **2013**, *1*, e26204. DOI: 10.4161/idp.26204

12 Bokor, M.; Tantos, Á.; Mészáros, A.; Jenei, B.; Haminda, R.; Tompa, P.; Tompa, K. Molecular Motions and Interactions in Aqueous Solutions of Thymosin-β_4, Stabilin CTD and Their 1 : 1 Complex, Studied by ^{1}H-NMR Spectroscopy. *Chemphyschem.* **2020**, *21*, 1420-1428. DOI: 10.1002/cphc.202000264

13 Kovács, D.; Kalmár, É.; Török, Z.; Tompa, P. Chaperone activity of ERD10 and ERD14, two disordered stress-related plant proteins. *Plant Physiol.* **2008**, *147*, 381–390. DOI: 10.1104/pp.108.118208

14 Murvai, N.; Kalmár, L.; Szabó, B.; Schád, É.; Micsonai, A.; Kardos, J.; Buday, L.; Han, K.–H.; Tompa, P.; Tantos Á. Cellular Chaperone Function of Intrinsically Disordered Dehydrin ERD14. *Int. J. Mol. Sci.* **2021**, *22*, 6190. DOI: 10.3390/ijms22126190
